# Effects of functional tasks exercise on cognitive functions of older adults with mild cognitive impairment: a randomized controlled pilot trial

**DOI:** 10.1186/s13195-019-0548-2

**Published:** 2019-12-04

**Authors:** Lawla L. F. Law, Vincent C. T. Mok, Matthew M. K. Yau

**Affiliations:** 1 School of Medical and Health Sciences, Tung Wah College, Block A, 98 Shantung Street, Mongkok, Hong Kong SAR; 2 Therese Pei Fong Chow Research Centre for Prevention of Dementia, Gerald Choa Neuroscience Centre, Lui Che Woo Institute of Innovative Medicine, Division of Neurology, Department of Medicine and Therapeutics, Faculty of Medicine, The Chinese University of Hong Kong. Prince of Wales Hospital, 30–32 Ngan Shing Street, Shatin, New Territories Hong Kong SAR; 3 School of Medical and Health Sciences, Tung Wah College, 31 Wylie Road, Homantin, Hong Kong SAR

**Keywords:** Mild cognitive impairment, Functional tasks exercise, Randomized controlled trial, Caregiver burden

## Abstract

**Background:**

Dementia has been presenting an imminent public health challenge worldwide. Studies have shown a combination of cognitive and physical trainings may have synergistic value for improving cognitive functions. Daily functional tasks are innately cognitive demanding and involve components found in common exercise. Individuals with mild cognitive impairment may demonstrate difficulties with complex activities of daily living. Functional tasks could possibly be used as a means of combined cognitive and exercise training for improving cognitive functions. This pilot aims to validate the effects of functional tasks exercise on cognitive functions and functional status in older adults with mild cognitive impairment.

**Methods:**

A four-arm, rater-blinded randomized controlled trial. Participants (*N* = 59) were randomized to either a functional task exercise group, a cognitive training group, an exercise training group, or a waitlist control group for 8 weeks. All outcome measures were undertaken at baseline and post-intervention using Neurobehavioral Cognitive Status Examination, Trail Making Test A and B, Chinese Version Verbal Learning Test, Lawton Instrumental Activities of Daily Living Scale, and Zarit Burden Interview.

**Results:**

Results of the Kruskal-Wallis one-way ANOVA showed higher improvement in the functional task exercise group with significant between-group differences in memory (*p* = 0.009) compared to the exercise group and cognitive training group, functional status (*p* = 0.005) compared to the cognitive training group and waitlist control group, and caregiver burden (*p* = 0.037) compared to the exercise group and cognitive training group.

**Conclusion:**

This pilot study showed that functional tasks exercise using simulated functional tasks as a means of combined cognitive and exercise program is feasible and beneficial in improving the memory and functional status of older adults with mild cognitive impairment as well as reducing the care-related burdens of their caregivers. The present findings warrant further well-designed longitudinal studies to examine the sustainability of effects and draw more definitive conclusions.

**Trial registration:**

Australian New Zealand Clinical Trials Registry, ACTRN 12616001635459. Registered on 25 November 2016.

## Background

Dementia is one of the most disabling conditions in older adults, impacting on the individuals and their families as well as imposing huge burden on societal and health care systems [1]. In the World Alzheimer Report 2015, it was estimated that global prevalence of dementia was about 47 million, with a 30% increase comparing to the estimate for 2010, and this number was projected to further increase to 75 million by 2030 and 132 million by 2050. The worldwide cost of dementia was $818 billion in 2015 and is expected to rise massively to $2 trillion by the year 2030 [2]. The World Health Organization (WHO) desperately called for global actions to tackle this imminent public health challenge in the First WHO Ministerial Conference in 2015, emphasizing the urgent need for accelerating the discovery of interventions to delay the onset and/or slow the progression of dementia [3]. Any interventions which could delay the onset of dementia by 5 years would reduce 57% of people with dementia [4]. Even a modest 1-year delay in the onset of dementia could give an 11% reduction in the prevalence [5].

With recognition of the implausibility of developing a cure or effective treatment for dementia within a decade [6], mild cognitive impairment (MCI), the prodromal stage of dementia, becomes an area of particular interest in research [7, 8]. Individuals with MCI are at increased risk of progressing to Alzheimer’s diseases and other dementias, with an annual conversion rate of 12–15% compared with 1–2% in their healthy peers [9] and this may even increase up to 50% in 2–3 years [10]. Nevertheless, individual with MCI may continue functioning without progressing to dementia or even revert and improve in their cognition and daily functions [11]. It is highly possible to delay the onset of dementia through interventions by slowing the rate of cognitive decline or improving the cognitive functions of those with MCI.

The beneficial effects of physical exercise on cognitive functions have been well-recognized [12]. Similarly, in light of the positive benefits of adopting an active lifestyle and participating in mentally stimulating activities for promoting cognitive vitality, the potential benefits of cognition-based interventions in population with MCI have also been explored [13]. Notably, studies found that combined cognitive and physical activities could induce a greater increase in neurogenesis [14, 15]. A combination of cognitive and physical trainings may have synergistic value for improving cognitive functions. Report from a recent systematic review has supported the potential beneficial effects of combined cognitive and exercise intervention on cognitive functions and functional status in persons with MCI [16]. Individuals with MCI may have difficulties in performing complex activities of daily living [17]. Daily functional tasks, such as cleansing or shopping, are innately cognitive demanding and involve components found in common exercise such as stretching, strengthening, endurance, and balance. A structured functional task exercise program, using simulated functional tasks as a means of combined cognitive and exercise intervention was developed and with the details being reported [18]. Simulated functional tasks of object placing and collection (cups and bowls) following specific patterns of movement and sequence are used and sit-stand movements are incorporated in the program. The program has five levels including unilateral movement, bimanual movement, task switching, and body midline crossing. A brief description of the five levels of movement is illustrated in the Appendix. The initial findings in our previous study have shown beneficial effects of the functional task exercise program in improving the cognitive functions and functional status of older adults with MCI [19]. However, the previous study did not include an exercise-only or a no-treatment comparison group, which can enable a better understanding of the overall significance of the program. This present pilot aims to examine the feasibility of conducting a four-armed randomized controlled trial (RCT) to further validate the effects of functional tasks exercise on cognitive functions and functional status in older adults with MCI. The hypothesis is that simulated functional tasks can be used as a means of combined cognitive and exercise intervention to influence different cognitive domains and improve cognitive functions and functional status of older adults with MCI.

## Methods

### Study design

The pilot was a four-arm, single-blind (rater-blinded) randomized controlled trial. After baseline assessment, all participants were randomized to either a functional task exercise group, a cognitive training group, an exercise training group, or a waitlist control group according to a list of computer-generated random numbers. All of the participants continued with their usual medical care. Ethics approval for this study was obtained from the Hospital Authority Research Ethics Committee. Written informed consent was obtained from all participants.

### Participants

The pilot was conducted from March 2017 to April 2018 at a local outpatient clinic and a community center in Hong Kong. Older adults (age 60+) with mild cognitive decline living in community were eligible for the study if they met the inclusion criteria for MCI [20]: (1) memory/cognitive complaint as reported by the patients or the caregivers, (2) objective cognitive impairment in one or more domains as revealed by neuropsychological assessment, but with (3) intact personal self-care functions, and (4) no confirmed diagnosis of dementia. The exclusion criteria were the following: (1) history of brain lesion/psychoactive substance abuse/comorbid medical conditions associated with cognitive/functional decline, (2) clinically significant depression, (3) known psychiatric cause of cognitive dysfunction, (4) medical conditions which rendered patients unable to engage in physical activity, (5) taking medications with significant impacts on cognitive function, and (6) significant impairment of vision, hearing, or communication that might affect participation in the assessments or program.

### Measurements

All outcome assessments were conducted at baseline and post-intervention by independent assessors. Primary outcomes were the Neurobehavioral Cognitive Status Examination (NCSE) for general cognitive function [21], Chinese Version Verbal Learning Test (CVVLT) for memory [22], and Trail Making Test A (TMT-A) and Trail Making Test B (TMT-B) for executive function [23]. Secondary outcomes were the Lawton Instrumental Activities of Daily Living Scale (Lawton IADL) for functional status [24] and the Zarit Burden Interview (ZBI) for caregiver’s burden [25]. To summarize the general cognitive performance, apart from the NCSE composite score calculated by adding all subtest scores (maximum 82), a NCSE normal domains score (0–10) was calculated by adding the number of domains with normal scores [26].

### Interventions

#### Functional task exercise group

The functional tasks exercise involved a total of 12 sessions in a group of 4–6 for 8 weeks, facilitated by an occupational therapist. All sessions began with a 5–10-min warm-up, followed by a 30–40-min core functional tasks exercise, and ended with a 5–10-min cool-down. Repetitions and activity speed were progressed according to the ability and comfort level of individual participants.

#### Cognitive training group

The cognitive training group received an existing center-based computer cognitive training program for training of attention, memory, executive function, and visual perceptual function in a group of 4–6 (60-min session; total 12 sessions) supervised by an occupational therapist for a total of 8 weeks.

#### Exercise training group

The exercise training group performed 12 sessions of exercise (60-min session) in group of 4–6, facilitated by an occupational therapist and an assistant for a total of 8 weeks. All exercise sessions began with a 5–10-min warm-up of light stretching to increase flexibility, 30–40-min moderate intensity aerobic exercise, including structured whole body movement exercise, bicycle and arm ergometry, at 4–5/10 on rate of perceived exertion and ended with a 5–10-min cool-down.

#### Waitlist control group

Participants in the control group were advised to maintain their normal activity or exercise pattern during the 8-week intervention period.

All the participants in each group continued with their routine medical care.

#### Statistical analysis

All analyses were performed using SPSS 23 (SPSS, Inc., Chicago, IL, USA). Group differences in demographics and all outcome measures at baseline were compared using Kruskal-Wallis test and Fisher’s exact test when appropriate.

Wilcoxon signed-rank tests were performed to evaluate the intervention effect (within-group) by time from baseline to post-intervention. Kruskal-Wallis tests were conducted to evaluate the between-group differences at post-intervention for the four groups on delta score (post-intervention score – baseline score) of all outcomes. Dunn’s pairwise tests and post hoc Bonferroni correction were carried out for all measures when significant between-group differences were revealed. Data were analyzed according to the intention-to-treat principle. Missing data for participants were replaced by the last available data (last observation carried forward). The statistic significant level was set at *p* < 0.05 (two-tailed).

## Results

### Participant characteristics

**Fig. 1:**
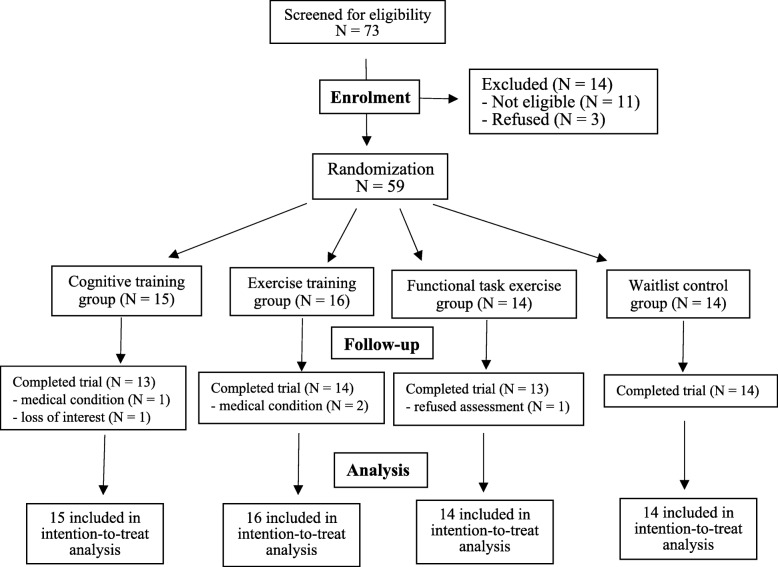
Study flow chart

A total of 73 potential participants were screened for eligibility. Figure 1 shows the flow of participants. Fifty-nine participants (35 females and 24 males), aged 60–89 years (mean = 75.5, SD = 7.45), were randomized into the functional task exercise group (*n* = 14), the cognitive training group (*n* = 15), the exercise training group (*n* = 16), or a waitlist control group (*n* = 14). Baseline characteristics are tabulated in Table 1. No significant baseline differences were found in demographic characteristics (range *p* = 0.250–0.946) or neuropsychological assessment results (range *p* = 0.133–0.936) between the four groups.

**Table 1:** Baseline demographic and clinical characteristics of participants

Characteristics	Cognitive training group (*n* = 15)	Exercise training group (*n* = 16)	Functional task exercise group (*n* = 14)	Waitlist control group (*n* = 14)	*p* value
Age, years^a^, [range/mean (SD)]	62–86/76.93 (6.79)	68–88/77.94 (6.11)	64–85/71.57 (7.43)	60–89/75.14 (8.53)	0.250
Gender^a^, *n* (%), (female/male)	8 (53.3)/7 (46.7)	8 (50)/8 (50)	10 (71.4)/4 (28.6)	9 (64.3)/5 (35.7)	0.643
Education level^a^, *n* (%), (illiterate/primary/secondary/tertiary)	4 (26.7)/6 (40)/5 (33.3)	5 (31.3)/3 (18.8)/7 (43.8)	4 (28.6)/5 (35.7)/4 (28.6)/1 (7.1)	3 (21.4)/7 (50)/3 (21.4)/1 (7.1)	0.772
Social status^a^, *n* (%), (living with family/alone)	9 (60)/6 (40)	12 (75)/4 (25)	12 (85.7)/2 (14.3)	12 (85.7)/2 (14.3)	0.365
Exercise per day^a^, *n* (%), (0/< 30 min/> 30 min)	3 (20)/3 (20)/8 (53.3)	3 (18.8)/4 (25)/9 (56.3)	3 (21.4)/4 (28.6)/7 (50)	1 (7.1)/5 (35.7)/8 (57.1)	0.946
Ambulatory level^a^, *n* (%), (unaided/with stick)	13 (86.7)/2 (13.3)	13 (81.3)/3 (18.8)	10 (71.4)/4 (28.6)	13 (92.9)/1 (7.1)	0.512
NCSE domain normal^b^, [mean (SD)]	6.8 (1.78)	7.0 (2.03)	7.50 (1.74)	6.79 (1.48)	0.510
NCSE composite score^b^, [mean (SD)]	61.27 (9.24)	61.19 (13.54)	65.78 (9.34)	62.14 (8.46)	0.622
CVVLT total free recall^b^, [mean (SD)]	14.93 (3.95)	15.50 (4.70)	15.93 (6.84)	15.00 (4.88)	0.936
CVVLT 30 s delayed recall^b^, [mean (SD)]	2.87 (2.13)	3.50 (1.86)	4.07 (2.09)	4.57 (1.65)	0.133
CVVLT 10 min delayed recall^b^, [mean (SD)]	1.60 (1.96)	2.19 (2.07)	3.21 (2.49)	2.57 (2.41)	0.288
TMT-A, seconds^b^, [mean (SD)]	122.87 (80.96)	126.19 (89.48)	96.21 (45.53)	115.71 (66.75)	0.826
TMT-B, seconds^b^, [mean (SD)]	238.93 (89.79)	225.25 (77.46)	188.86 (92.64)	202.35 (82.91)	0.411
Lawton IADL^b^, [mean (SD)]	17.93 (5.71)	17.81 (4.56)	20.42 (2.50)	18.64 (4.94)	0.391
ZBI^b^, [mean (SD)]	25.2 (9.65)	28.67 (10.97)	18.83 (11.37)	25.00 (8.84)	0.340

*NCSE* Neurobehavioral Cognitive Status Examination, *CVVLT* Chinese Version Verbal Learning Test, *TMT-A* Trail Making Test-part A, *TMT-B* Trail Making Test-part B, *Lawton IADL* Lawton Instrumental Activities of Daily Living Scale, *ZBI* Zarit Burden Interview

^a^Fisher’s exact test

^b^Kruskal-Wallis test

### Compliance

Of the 59 participants who completed the baseline assessment, 54 (91.5%) participants completed the assigned programs and performed the post-intervention evaluation. Dropout rates did not vary significantly between the groups at post-intervention (*p* = 0.742, two-tailed Fisher’s exact test). No adverse events were reported from any of the groups.

### Outcomes

Performance of the four groups for all outcome measures and the between-group comparisons are illustrated in Table 2.

**Table 2:** Outcome comparisons at baseline and post-intervention

Measures	Cognitive training group (*n* = 15)			Exercise training group (*n* = 16)			Functional task exercise group (*n* = 14)			Waitlist control group (*n* = 14)			*p* value (group)
	Pre-test mean (SD)	Post-test mean (SD)	Gain score mean (SD) (95% CI)	Pre-test mean (SD)	Post-test mean (SD)	Gain score mean (SD) (95% CI)	Pre-test mean (SD)	Post-test mean (SD)	Gain score mean (SD) (95% CI)	Pre-test mean (SD)	Post-test mean (SD)	Gain score mean (SD) (95% CI)	
NCSE domain normal	6.8 (1.78)	6.40 (2.20)	− 0.40 (1.24) (− 1.08–0.28)	7.0 (2.03)	6.94 (1.95)	− 0.06 (1.12) (− 0.66–0.53)	7.50 (1.74)	7.57 (1.78)	0.07 (1.07) (− 0.54–0.69)	6.79 (1.48)	6.43 (1.74)	− 0.35 (1.15) (− 1.02–0.30)	0.653
*p* value^#^	0.223			0.873			0.803			0.272			
NCSE composite score	61.27 (9.24)	58.80 (11.01)	− 2.46 (5.69) (− 5.61–0.68)	61.19 (13.54)	60.69 (11.33)	− 5.00 (6.02) (− 3.70–2.70)	65.78 (9.34)	66.14 (9.71)	0.35 (4.89) (− 2.46–3.18)	62.14 (8.46)	59.57 (8.46)	− 2.57 (4.05) (− 4.91 to − 0.23)	0.785
*p* value^#^	0.153			0.660			0.789			0.033*			
CVVLT total free recall	14.93 (3.95)	14.80 (5.45)	− 1.33 (4.14) (− 2.42–2.15)	15.50 (4.70)	13.69 (5.52)	− 1.81 (5.94) (− 4.97–1.35)	15.93 (6.84)	20.57 (6.74)	4.64 (4.67) (1.94–7.33)	15.00 (4.88)	16.43 (4.85)	1.43 (3.11) (− 0.36–3.22)	0.009**
*p* value^#^	0.893			0.241			0.006**			0.103			
CVVLT 30s delayed recall	2.87 (2.13)	3.60 (1.96)	0.73 (2.09) (− 0.42–1.88)	3.50 (1.86)	2.94 (2.02)	− 0.56 (2.16) (− 1.71–0.58)	4.07 (2.09)	5.28 (2.73)	1.21 (1.88) (0.12–2.30)	4.57 (1.65)	4.29 (2.55)	− 0.28 (1.54) (− 1.17–0.60)	0.096
*p* value^#^	0.182			0.286			0.038*			0.433			
CVVLT 10 min delayed recall	1.60 (1.96)	2.07 (2.02)	0.47 (1.64) (−0.44–1.37)	2.19 (2.07)	2.00 (2.07)	− 0.19 (1.38) (− 0.92–0.54)	3.21 (2.49)	4.78 (2.99)	1.57 (1.95) (0.44–2.69)	2.57 (2.41)	2.93 (2.84)	0.36 (1.86) (− 0.71–1.43)	0.071
*p* value^#^	0.131			0.469			0.015*			0.340			
TMT-A	122.87 (80.96)	126.13 (83.71)	3.27 (67.41) (− 34.06–40.59)	126.19 (89.48)	126.00 (77.51)	− 0.188 (30.17) (− 16.26–15.89)	96.21 (45.53)	70.36 (31.02)	− 25.14 (28.15) (− 41.39 to − 8.88)	115.71 (66.75)	116.50 (71.76)	0.79 (33.89) (− 18.78–20.35)	0.085
*p* value^#^	0.937			0.889			0.006**			0.695			
TMT-B	238.93 (89.79)	235.60 (86.67)	− 2.67 (31.40) (− 20.05–14.72)	225.25 (77.46)	213.69 (93.16)	− 11.56 (63.61) (− 45.45–22.33)	188.86 (92.64)	155.71 (98.17)	− 33.14 (56.97) (− 66.03 to − 0.25)	202.35 (82.91)	225.43 (80.24)	1.07 (57.63) (− 32.20–34.5)	0.095
*p* value^#^	1.000			0.674			0.023*			0.139			
Lawton IADL	17.93 (5.71)	17.80 (6.11)	− 0.13 (2.45) (− 1.48–1.22)	17.81 (4.56)	18.50 (3.98)	0.69 (2.15) (− 0.45–1.83)	20.42 (2.50)	22.50 (3.13)	2.07 (1.77) (1.04–3.09)	18.64 (4.94)	18.14 (4.87)	− 0.50 (1.83) (− 1.55–0.55)	0.005**
*p* value^#^	1.000			0.287			0.005**			0.234			
ZBI	25.2 (9.65)	38.40 (27.56)	13.20 (23.78) (− 16.33–42.73)	28.67 (10.97)	30.56 (12.89)	1.89 (6.86) (− 3.38–7.16)	18.83 (11.37)	13.67 (13.92)	− 5.16 (6.74) (− 12.23–1.90)	25.00 (8.84)	24.38 (9.43)	− 0.63 (1.19) (− 1.61–0.36)	0.037*
*p* value^#^	0.285			0.655			0.042*			0.180			

*CI* confidence interval, *NCSE* Neurobehavioral Cognitive Status Examination, *CVVLT* Chinese Version Verbal Learning Test, *TMT-A* Trail Making Test-part A, *TMT-B* Trail Making Test-part B, *Lawton IADL* Lawton Instrumental Activities of Daily Living Scale, *ZBI* Zarit Burden Interview

*p* value^#^ = within-group effects (by time): *p < 0.05; **p < 0.01

*p* value (group) = between-group effects: *p < 0.05; **p < 0.01

The results of Wilcoxon signed-rank tests showed that the functional task exercise group demonstrated significant within-group improvements in all outcomes (*p* range = 0.006–0.042) except general cognitive function at post-intervention. The waitlist control group showed a significant decrease in the NCSE composite score while the number of normal domains in general cognitive function did not show any significant within-group changes. Both the cognitive training group and the exercise training group did not show any significant within-group differences.

At post-intervention, results of the Kruskal-Wallis test showed that there were significant between-group differences in memory as revealed by the CVVLT total free recall score (*χ*^2^ (3) = 11.58, *p* = 0.009), functional status as revealed by the Lawton IADL score (*χ*^2^ (3) = 13.04, *p* = 0.005), and caregiver’s burden as revealed by the ZBI score (*χ*^2^ (3) = 8.50, *p* = 0.037). The results also showed an approaching significant difference in executive function as revealed by the TMT-A score (*χ*^2^ (3) = 6.62, *p* = 0.085) and TMT-B score (*χ*^2^ (3) = 6.38, *p* = 0.095).

Post hoc Dunn’s pairwise tests were conducted with Bonferroni correction. The performance of functional task exercise group in memory (mean rank = 42.04) was significantly higher compared to the exercise group (mean rank = 22.41; *p* = 0.002), and the cognitive training group (mean rank = 25.10; *p* = 0.007). The functional task exercise group (mean rank = 42.93) was also significantly different to the waitlist control group (mean rank = 21.18; *p* = 0.001), the cognitive training group (mean rank = 25.97; *p* = 0.007), and the exercise group (mean rank = 30.19; *p* = 0.038) in functional status. The caregiver’s burden was also found significantly lower in the functional task exercise group (mean rank = 7.08) compared to the exercise group (mean rank = 17.11; *p* = 0.01) and the cognitive training group (mean rank = 18.40; *p* = 0.012) as well as an approaching significant difference compared to the waitlist control group (mean rank = 14.69; *p* = 0.058). There was no evidence of a significant difference between the other pairs.

## Discussion

The aim of the present pilot was to examine the feasibility of conducting a four-armed comparison to validate the effects of a functional tasks exercise on cognitive functions and functional status in older adults with MCI. This pilot demonstrated the feasibility of acquiring good compliance (91.5%) of participants randomized into the intervention and comparison groups as compared to the average compliance of 85% reported in a review on similar studies [27]. Interestingly, the waitlist control group demonstrated the highest compliance (100%) among the four groups and the group did not show significantly lower performance compared to the cognitive training group or the exercise training group in any outcome measures. Studies have shown motivational abilities of persons with MCI, which include the ability to decide to do a specific task and the ability to stay with the task, may have protective influence on their cognitive decline and are associated with the stability of MCI [28, 29]. Further studies including psychological outcomes such as motivational abilities might help better understand the potential contributing factors involved. At post-intervention, the functional task exercise group showed significantly greater improvement in memory and caregiver burden compared to the exercise training only and the cognitive training only groups as well as in the functional status compared to the cognitive training only and the waitlist control groups.

The findings of this study support previous similar studies demonstrating that the combined cognitive and exercise training group outperformed the single-component counterparts or control groups [16, 30, 31] although comparable studies on the differential effects in persons with MCI are still limited [32]. The demonstrated advantage of combined exercise and cognitive training over single-component training could be ascribed to the potential additive effects on neurogenesis resulting from the initial pro-proliferative primed by the exercise component and the subsequent survival-promoting effects induced by the cognitive challenges from the cognitive component [33, 34]. The cognitive component also plays a crucial role in guiding the newly generated neurons through an activity-dependent synaptic adaptation for functional integration into the network of the working brain which further leads to lasting positive plastic changes and improves the cognitive functions [35, 36]. The functional task exercise group showed significant between-group improvement in memory. In the present study, the cognitive component of functional tasks exercise involves spatial tasks of object placing and collection following specific patterns. Studies have shown a combination of exercise and hippocampus-dependent learning tasks such as spatial tasks can enhance hippocampal neurogenesis [37, 38]. Therefore, the performance of the spatial tasks in functional tasks exercise could possibly enhance neurogenesis in the hippocampus, which is an important brain area for learning and memory, and contribute to the improvement in memory.

Indeed, memory is an important cognitive correlate of everyday functional abilities [39]. In particular, the ability to remember the order of items or events in sequence contributes uniquely to everyday functioning [40]. Activities of daily living comprise different order of actions and events which require sequencing ability to allow integrative performance and meaningful experiences, for example, getting ready to work after getting up from bed, having breakfast, and taking transport to office; or preparing meats and vegetables for cooking and turning off the gas stove at planned time when finish in meal preparation. The ability to temporally sequence events and to produce purposeful actions in an effective order is critical for organization and successful completion of everyday tasks. However, deficits in sequencing ability or temporal order memory has been found in persons with MCI and associated with decline in instrumental activities of daily living [41, 42]. Propitiously, sequencing ability can be trained, and the training gain can be transferred to untrained tasks [43]. The functional tasks exercise used in the present study involves a component of object placing and collection in forward and backward sequence respectively. Performance of this motor sequence task can exert high cognitive demand for retrieval and manipulation of online information while simultaneously maintaining the memory of object position in place and producing a sequence of goal-directed movement [18]. Successful performance of the motor sequence tasks in the functional tasks exercise could possibly contribute to the improvement in sequencing ability and thus enhancing the functional status demonstrated in the functional tasks exercise group. Studies also suggested that the generalization effects of sequence training to untrained tasks could be related to implicit learning of stimulus non-specific structure during the process which would facilitate prediction in future events [44, 45]. Practice of sequence motor tasks or sequence learning can also activate hippocampus which is not only associated with learning and memory but also an important area supporting the translation of meaningful experience into adaptive behavior for successful interactions in impending future [43, 44, 46] and thus enhancing performance in everyday functions.

Importantly, the functional task exercise group demonstrated significant between-group reduction in caregiver burden which is seldom included or reported in similar studies [32]. Although persons with MCI are still independent in most of the activities of daily living, more than 30% of caregivers of persons with MCI report experiencing a clinically significant burden [47] and their needs for support services are found comparable to caregiver of persons with Alzheimer’s disease [48]. Caregiver burden not only can have an adverse impact on the caregiver’s mental and physical well-beings, but it is also strongly associated with early institutionalization of their relatives or friends being cared [49]. Functional decline and increasing dependence of persons with MCI have been found being the most predictive marker for the burden increase of the caregiver while cognitive symptoms may only impose an increasing demand at a later stage of disease progression [50]. Therefore, it is plausible that the improvement in functional status of the functional task exercise group may contribute to the reduction in caregiver burden of the group.

This study differs from previous similar studies [30–32] with older adults in that this is a four-arm study in population with MCI comparing the intervention group with cognitive and exercise training alone groups and waitlist control group and with equal amount of training exposure. To the authors’ best knowledge, this is the first program that uses structured functional tasks as a means of combined cognitive and exercise intervention and compares the differential effects in persons with MCI. The present study has demonstrated the potential for use and acceptability of the functional tasks exercise in older persons with MCI.

### Limitations

Although the study results are encouraging, there are limitations that warrant mention. Firstly, the findings from the present study with a small sample will need to be further validated in future well-designed larger scale randomized controlled studies.

According to a power calculation, 34 participants in each group will be required for 80% power to detect a significant group difference of 5 points on memory test with a significance level of 0.05 [51]. Assuming a dropout rate of 15%, a total of 160 participants will be needed for four groups in future studies.

Secondly, the small sample size limits the control of potential confounding factors during the analysis and did not allow stratification of participants into different groups of age, education, or exercise pattern which may influence the intervention responses and affect the outcomes. Further, although the Trail Making Tests used in this study are common outcome measures for assessing executive function, these paper and pencil tests, especially TMT-B, can be difficult for elderly participants to complete. Including more ecologically valid executive function measures, such as everyday problem solving tests, could provide a more practical and comprehensive indication on participant’s level of everyday executive functions. Last but not least, inclusion of longitudinal follow-up assessments is needed to gain insight into the potential maintenance effects of the interventions.

## Conclusion

In conclusion, this pilot study showed that functional tasks exercise using simulated functional tasks as a means of combined cognitive and exercise program is feasible and beneficial in improving the memory and functional status of older adults with MCI as well as reducing the care-related burdens of their caregivers. The waitlist control group showed no significant difference compared to the cognitive training only and the exercise training groups. Further well-designed longitudinal studies with an adequate sample size are needed to examine the sustainability of effects and draw more definitive conclusions.

## Data Availability

The datasets of the current study are available from the corresponding author on reasonable request.

## Appendix

**Table 3:** Five levels of key movements in functional tasks exercise

Level	Basic movement pattern
Level 1	Simple place/collection. Cross midline reaching. Forward placing and backward collection.
Level 2	Circular place/collection. Cross midline reaching. Clockwise placing and counterclockwise collection.
Level 3	Alternate place/collection. Cross midline reaching. Place/collect with left/right hand alternatively.
Level 4	Repeated place/collection. Cross midline reaching. Place/collect with 1 point of repetition.
Level 5	Bimanual alternate place/collection. Cross midline reaching. Place/collect bimanually in opposite directions.

## Abbreviations

ANOVA: Analysis of variance
CVVLT: Chinese Version Verbal Learning Test
Lawton IADL: Lawton Instrumental Activities of Daily Living Scale
MCI: Mild cognitive impairment
NCSE: Neurobehavioral Cognitive Status Examination
TMT-A: Trail Making Test-part A
TMT-B: Trail Making Test-part B
WHO: World Health Organization
ZBI: Zarit Burden Interview
